# How grit enhances physical exercise in college students: mediating roles of personal growth initiative and self-efficacy

**DOI:** 10.3389/fpsyg.2025.1652984

**Published:** 2025-09-09

**Authors:** Chang Hu, Wen Zhang, Wenying Huang, Chanjuan Jin

**Affiliations:** ^1^Physical Education College, Jiangxi Normal University, Nanchang, China; ^2^College of Physical Education, Anhui University of Finance and Economics, Bengbu, China

**Keywords:** physical exercise, grit, personal growth initiative, self-efficacy, chain mediating effects

## Abstract

**Objective:**

This study aims to investigate the impact of Grit on physical exercise (PE) among college students and explore the underlying mechanisms, particularly focusing on the mediating roles of personal growth initiative (PGI) and self-efficacy (SE). It also examines how these psychological factors interact to influence physical activity, offering insights for educational interventions.

**Methods:**

A survey was conducted among 2,559 college students using the Short Grit Scale (Grit-S), the Personal Growth Initiative Scale-II (PGIS-II), the General Self-Efficacy Scale (GSE), and a single-item measure of physical exercise (PE).

**Results:**

(1) Grit, PGI, SE, and PE were significantly positively correlated with each other. (2) PGI and SE played significant mediating roles in the relationship between Grit and PE. Specifically, three mediating pathways were identified: the independent mediating effect of PGI, the independent mediating effect of SE, and the chain mediating effect of PGI and SE.

**Conclusion:**

Grit significantly influences PE among college students through the mediating effects of PGI and SE. Enhancing these psychological factors can be an effective strategy for increasing PE participation. This study provides a theoretical basis for developing targeted interventions to promote PE among college students.

## 1 Introduction

Survey data shows that globally, fewer than 30% of individuals meet the recommended physical activity guidelines (Singh et al., 2020; Tapia-Serrano et al., 2022; Garcia-Hermoso et al., 2023). This trend is particularly pronounced among Chinese college students, who often fail to meet these guidelines (Li et al., 2024). Given China's large student population, addressing this issue here could have significant global implications. Empirical research indicates that the proportion of Chinese college students meeting the recommended physical activity levels is significantly lower than the global average, with over 80% failing to meet the guidelines (Zhang et al., 2023). A systematic review of relevant literature also confirmed this phenomenon, clearly pointing out that the overall physical health of Chinese college students is on a downward trend, mainly manifested in a year-on-year decrease in exercise (Pan et al., 2022). The lack of adequate physical exercise (PE) is not only closely associated with numerous physical health risks, such as hypertension, coronary heart disease, hyperlipidemia, and fatty liver (De Ferrari et al., 2019; Kraus et al., 2019; Lee et al., 2021; Crea, 2022), but also linked to a variety of mental health issues, including depression, sleep disorders, and anxiety (Kansagra, 2020; DelRosso et al., 2021; Pearce et al., 2022; Singh et al., 2023). Recent studies have shown that over 40% of college students report significant symptoms of depression and anxiety, which are often exacerbated by a sedentary lifestyle (Gao et al., 2021), a particularly concerning phenomenon. The issue is further compounded by the fact that college students are still in their developmental years, both physically and mentally, and habits formed during this period are likely to persist throughout their lives. Therefore, increasing PE levels among college students is crucial for improving their overall health and wellbeing.

While previous research has identified numerous barriers to PE, such as lack of time and resources (Terra et al., 2023; Alhroub et al., 2024), these models often overlook the critical role of positive psychological attributes that enable students to overcome such obstacles. Many earlier frameworks have focused more on prohibitive factors rather than on proactive traits that foster sustained engagement, leaving a significant gap in our understanding (Millstein et al., 2020). Given that the benefits of PE, such as enhanced mood and resilience, require sustained effort and persistence, psychological traits like Grit become critically important. Grit, which encompasses sustained passion and perseverance for long-term goals, has gained recognition in the field of positive psychology (Hill et al., 2016). It is closely tied to Subjective Wellbeing and overall development (Hou et al., 2022; Kannangara et al., 2018). PE is a key health-related behavior that boosts fitness, alleviates stress, and enhances mood. Given that higher Grit is linked to more frequent and intense PE in college students (De La Cruz et al., 2021; Hull and Vultaggio, 2019; Kim, 2020), it is crucial to delve deeper into how Grit influences exercise behaviors and the underlying psychological mechanisms. This line of inquiry can offer valuable insights for developing effective interventions to promote physical activity among college students.

Existing research has unveiled a correlation between Grit and PE frequency/intensity, but the pathways through which Grit exerts its influence remain elusive (Gorin et al., 2024). However, the majority of this research has focused on the direct relationship, leaving the underlying psychological process largely unexplored. This constitutes a significant conceptual gap: we know that gritty individuals exercise more, but we do not know why or how. Our study moves beyond a simple correlational analysis by proposing and testing a novel chain mediation model. We argue that grit does not operate in a vacuum; instead, it initiates a specific cognitive-motivational sequence. To address this gap, this study proposes and tests a chain mediation model. We hypothesize that personal growth initiative (PGI) and self-efficacy (SE) are pivotal in this process. Grit may foster PGI, which subsequently bolsters SE, and these two factors may serve as mediators in the relationship between Grit and PE. Therefore, the primary objective of this study is to investigate the chain mediating role of personal growth initiative and self-efficacy in the relationship between grit and physical exercise among college students.

## 2 Theory and hypothesis

### 2.1 Grit and physical exercise

In line with Caspersen et al., we differentiate general physical activity, which encompasses any bodily movement, from PE. PE is defined as a subset of physical activity that is planned, structured, and repetitive, with the ultimate goal of enhancing or maintaining physical fitness (Caspersen et al., 1985; Dasso, 2019). Sustaining intentional health behaviors like PE requires more than just an awareness of its benefits; it necessitates substantial psychological determination (Duckworth et al., 2007).

College students' participation in PE is influenced by a variety of factors, among which personal psychological traits, especially Grit, play a key role (Messina et al., 2020). To understand why some students maintain regular participation in PE while others do not, it is essential to examine the role of psychological traits such as Grit. Grit, characterized by the sustained passion and perseverance needed to achieve long-term goals (Guelmami et al., 2022), is a fundamental trait in this context. Motivation and social cognitive theory (Schunk and DiBenedetto, 2020) provides a framework for understanding how such traits influence behavior. Within this framework, Grit can be conceptualized as a decisive personal factor that drives an individual's agency and determination, thereby influencing their behavioral choices and their persistence in the face of environmental or self-regulatory challenges in the exercise domain (Gifford and Nilsson, 2014).

As an important psychological resource, grit can directly translate to PE-related behaviors, such as maintaining a consistent workout frequency (e.g., exercising 3-4 times per week), increasing the duration or intensity of sessions over time, and persisting with the exercise regimen despite academic stress or lack of motivation (Liu M. et al., 2024; Longakit et al., 2025). The positive impact of Grit on healthy behaviors is mainly reflected in two aspects: mental health and behavioral participation. On the one hand, individuals with high Grit can maintain an optimistic and open-minded attitude when facing life's stress and setbacks, and are less easily swayed by negative emotions (Hou et al., 2022; Zhou et al., 2025). On the other hand, individuals with high Grit exhibit higher self-discipline and proactivity in adopting healthy behaviors, effectively adhering to habits such as regular exercise and a balanced diet (Totosy De Zepetnek et al., 2021). Drawing from the preceding discussion, the present research puts forward a hypothesis:

**H1:** Grit is positively correlated with college students' PE.

### 2.2 The mediating role of personal growth initiative

The Organic Assessment Process Theory posits that individuals consciously evaluate the potential value of novel stimuli for their growth and self-actualization (Rogers, 1964), thereby influencing their decisions. In simpler terms, this theory suggests that people have an innate drive to grow and improve, and they actively seek out and engage in challenging activities that help them realize their full potential. PGI, which is the conscious and proactive tendency to improve oneself, is closely linked to self-development and behavioral choices (Cai and Lian, 2022; Gong et al., 2022). Those with a high degree of PGI possess an intense aspiration for self-improvement and are adept at identifying their developmental needs, planning actions, and actively implementing them (Feraco et al., 2023; Yue et al., 2025). In the context of physical exercise, for example, a student with high PGI might actively seek out new workout routines, set progressive fitness goals, and proactively schedule gym sessions into their weekly calendar. Research has shown that PGI helps establish healthy lifestyles and promotes comprehensive physical and mental development (Lin et al., 2024; Weigold et al., 2024).

Individuals with Grit, characterized by high goal commitment, are more likely to view regular exercise as a pathway for self-improvement when faced with PE, which promotes physical and mental health (Totosy De Zepetnek et al., 2021). PGI can further motivate these individuals to adjust strategies, overcome obstacles, and persistently achieve their exercise goals. Studies have demonstrated a significant correlation between Grit and PGI, with individuals who are highly gritty being more adept at breaking down long-term goals into phased plans and demonstrating stronger self-development motivation (Xu et al., 2023; Lam and Zhou, 2020; Styk et al., 2019). While this connection has been established primarily in academic and professional settings in Western cultures, we expect this relationship to hold within the Chinese university context, where both perseverance and the drive for self-improvement are highly valued cultural ideals. PGI is also a key driver of positive behavioral change, closely related to the persistence and intensity of PE (Shurtz et al., 2024). Students with high PGI are more likely to persist in their exercise. Thus, PGI complements grit in ensuring long-term adherence; while grit provides the staying power to endure challenges, PGI provides the strategic direction and motivation for continuous improvement, preventing stagnation. Based on this, the current study puts forward a hypothesis:

**H2:** PGI serves as a mediator in the relationship between Grit and PE among college students.

### 2.3 The mediating role of self-efficacy

Bandura's self-efficacy theory posits that an individual's subjective assessment of their capability to complete a specific task, namely SE, significantly influences behavioral choices, the effort invested, and perseverance in the face of challenges, making it a crucial factor for behavioral change and goal attainment (Bandura, 1997). Grit, characterized by persistent dedication and sustained effort toward long-term goals, is closely linked to SE (Meng et al., 2025; De Lorenco-Lima et al., 2025). Individuals with Grit often have high SE, setting challenging goals and taking proactive steps to achieve them (Dale and Yan, 2024; Hwang and Lee, 2024).

The Organic Assessment Process Theory suggests that a clear understanding of one's abilities helps evaluate the potential value of new stimuli, facilitating self-actualization (Maurer and Daukantaite, 2020; Li et al., 2023). For college students, engaging in PE, which enhances both physical and mental wellbeing, requires overcoming challenges such as inertia and effective time management (Snedden et al., 2019; Martín-Rodríguez et al., 2024). Students with high Grit are more likely to view physical activity as a means of self-improvement (Tao et al., 2024a). Their SE affects their confidence in maintaining exercise routines, and an individual's belief in their ability to exercise is crucial for their actual participation. For instance, in structured exercise programs, an individual's self-efficacy can predict whether they will attempt more challenging workouts, adhere to the program when faced with fatigue, and ultimately achieve their fitness goals (Tao et al., 2024b). Several recent studies have consistently demonstrated a positive relationship between grit and self-efficacy (Alhadabi and Karpinski, 2020; Wolters and Hussain, 2015; Özhan, 2021). Based on this, the present study proposes a hypothesis.

**H3:** SE serves as a mediator in the relationship between Grit and PE among college students.

### 2.4 Construction of the chain mediator model

According to social cognitive theory, an individual's cognitive factors and behaviors interact with each other, jointly shaping the occurrence and maintenance of behaviors (Bandura, 2001). Specifically, the PGI fuels the belief in one's capability to act SE. PGI reflects an individual's active pursuit of self-development, proactive goal-setting, and implementation of actions, while SE represents an individuals' subjective judgment of their ability to complete specific behaviors (Cai and Lian, 2022). Individuals with high PGI are likely to explore pathways for self-development actively. They set detailed, phased goals and feasible planning strategies, thereby accumulating successful experiences, which are vital sources for enhancing SE. For example, a student high in PGI who decides to improve their health will not only set a goal but will also create a detailed plan (e.g., researching running techniques, creating a weekly training schedule). This proactive planning, combined with the experience of early success in following the plan, directly builds their self-efficacy for the larger goal. Empirical studies have demonstrated a significant positive correlation between PGI and SE (Çankaya et al., 2017). Students' performance in planning, willingness to change, and proactive actions can positively predict their SE in specific tasks (Olckers and Harumavamwe, 2025). Conversely, students with low PGI may fail to transform their perseverance into tangible outcomes, even if they possess determination. This is due to the lack of effective goal-planning and action strategies, resulting in slow SE enhancement and even self-doubt (Jacobs et al., 1984; Cherewick et al., 2023; Sidenko, 2025). This insight suggests that psychological interventions aimed at boosting exercise adherence should not only focus on building self-efficacy directly but should also include components designed to foster a personal growth mindset.

**Figure 1:**
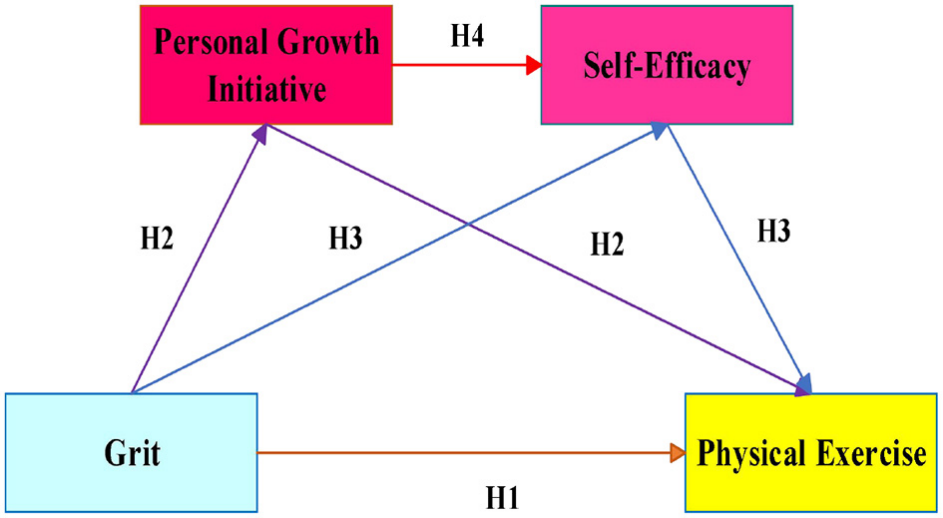
Hypothesis model diagram.

In the context of PE, Grit propels students to set long-term goals. Those with high PGI plan and tackle challenges, accumulating successful experiences that further enhance their SE. This sequential enhancement motivates their continued participation in PE. Therefore, PGI and SE may create a chain-mediation effect on exercise behavior (Meng et al., 2022; Kadier et al., 2025). Based on this, the study proposes a hypothesis (see Figure 1 for a visual representation of the proposed model):

**H4:** PGI and SE mediate the impact of Grit on PE among college students

In conclusion, this study focuses on Chinese college students to explore the relationship between Grit and PE. Additionally, the study examines whether PGI and SE mediate the relationship between Grit and PE. As illustrated in Figure 1, the study proposes a hypothetical model to uncover the underlying mechanisms through which Grit influences college students' PE. By exploring this psychological chain, this study not only offers a novel theoretical framework for understanding exercise adherence but also provides a foundation for developing targeted interventions that cultivate grit and its cognitive-motivational correlates to promote lifelong health.

## 3 Materials and methods

### 3.1 Participation

The sample size for this study was determined using GPower 3.1 software and empirical rules for social science research. A power analysis for multiple regression in a chain mediation model indicated a minimum of 193 participants, assuming a medium effect size (*f*^2^ = 0.15), α = 0.05, and power = 0.80 (Kang, 2021). Given the model's complexity, the number of predictors was set to 5. Empirical rules suggested a sample size of 10 to 15 times the number of questionnaire items (35 items), recommending 350 to 525 participants. Integrating these methods, a minimum sample size of 350 was established (Lakens, 2021). While a minimum sample size of 350 was established, we aimed to collect a substantially larger sample to increase statistical power, minimize sampling error, and enhance the generalizability and robustness of our findings within the Chinese college student population.

Data were collected from 2,559 college students from six colleges in central China between September 7 and November 3, 2024, using the online platform Wenjuanxing (https://www.sojump.com). Of the 2,716 responses collected, 2,559 were valid, yielding a 94.22% response rate. To ensure data quality, several measures were implemented. First, we excluded incomplete questionnaires. Second, we removed participants who took less than 3 min to complete the survey, as this indicated potentially careless or hasty responses.

Additionally, we excluded participants who showed a pattern of regular or repetitive answering, which suggested insincere or non-attentive participation. Third, two attention-check items (e.g., Please select ‘Strongly Agree' for this item) were embedded within the survey. Participants who failed to answer these items correctly were removed from the final dataset. This rigorous screening process helps ensure the reliability and validity of the self-reported data. The sample included 1,262 males (49.3%) and 1,297 females (50.7%), with an average age of 20.42 years (±2.55), and ages ranging from 17 to 25, which is typical for undergraduate students in China. The participants were recruited from six comprehensive universities in central China, which include natural sciences, engineering, arts, liberal arts, and engineering and technology, thereby representing a broad cross-section of the general Chinese college student population. The demographic details are presented in Table 1.

**Table 1:** Demographic and sample characteristics.

**Characteristics**	**Categories**	***N(%)***	***M ± SD***
Gender	Male	1,262 (49.3)	
	Female	1,297 (50.7)	
Family residence	Urban	1,069 (41.8)	
	Rural	1,490 (58.2)	
Subject major	Liberal Arts	833 (32.6)	
	Natural Sciences	528 (20.6)	
	Engineering and Technology	706 (27.6)	
	Arts	492 (19.2)	
Only child	No	2,047 (80.0)	
	Yes	512 (20.0))	
Age			20.42 ± 2.55

### 3.2 Measures

#### 3.2.1 The short grit scale (Grit-S)

This study employed the Grit-S scale to assess college students' Grit related to PE. Details of the items and scoring are provided in Table 2. Initially developed by (Duckworth and Quinn 2009), the Grit-S measures two dimensions: Consistency of Interest and Perseverance of Effort. It assesses individuals' passion and perseverance for long-term goals. Each dimension is evaluated through four items, scored on a five-point Likert scale from 1 (not at all like me) to 5 (very much like me).

**Table 2:** The short grit scale.

**Serial number**	**Measurement items**
Item 1	I find it easy to stick with my exercise routine, even when I don't feel like it.
Item 2	I don't give up on my exercise goals easily, no matter how tough it gets.
Item 3	I am dedicated to improving my fitness, even if it takes a long time.
Item 4	When I start a new exercise program, I always see it through to the end.
Item 5	I get excited about new exercise challenges and work hard to overcome them.
Item 6	I am able to maintain my motivation to exercise even when I face setbacks.
Item 7	I am consistent in my efforts to reach my fitness goals.
Item 8	I am always looking for ways to improve my physical performance.

To adapt the Grit-S scale for the context of physical exercise, we invited two psychology professors and two physical education professors to conduct a two-round content validity assessment of the scale. Their expertise ensured that the scale items were relevant and appropriate for measuring Grit in the context of physical exercise. Based on this, we conducted a pilot study involving 90 college students, with 88 participants providing valid data. The pilot study aimed to test the comprehensibility of the scale items and to conduct preliminary reliability and validity analyses. The results showed that the scale met the required psychometric standards.

Subsequently, we proceeded with the formal survey. In the formal survey, we first conducted an exploratory factor analysis (EFA). The EFA results revealed a KMO value of 0.889, with two factors having eigenvalues greater than 1, and the scree plot also indicated that a two-factor model was optimal. Building on this, we further conducted a confirmatory factor analysis (CFA). The CFA results showed good model fit indices, with χ^2^/*df* = 1.003, GFI = 0.998, AGFI = 0.996, TLI = 1, CFI = 1, and RMSEA = 0.001. In this study, the Cronbach's alpha coefficient was 0.849. These indices collectively demonstrated the scale's strong reliability and validity.

#### 3.2.2 Personal growth initiative scale-II (PGIS-II)

In this study, we employed the PGIS-II, devised by Robitschek et al. (2012), to measure college students' PGI. The scale includes 16 items that pertain to four core aspects: Readiness for Change, Planfulness, Utilization of Resources, and Intentional Behavior. Two illustrative items are: “I know how to set achievable goals for my exercise regimen” and “I recognize when it's time to make a specific change in my workout.” Respondents rate the items on a 6-point scale ranging from 0 (Strongly Disagree) to 5 (Strongly Agree). Higher cumulative scores on the PGIS-II indicate a stronger PGI. In this study, the PGIS-II demonstrated excellent internal consistency, with a Cronbach's alpha value of 0.753. Additionally, CFA supported the four-factor structure of the PGIS-II, showing good model fit indices: χ^2^/*df* = 1.627, GFI = 0.992, AGFI = 0.989, TLI = 0.990, CFI = 0.992, and RMSEA = 0.016. The scale has been widely used among Chinese students (Yue et al., 2025; Li et al., 2025), providing a reliable tool for researching their psychological growth and initiative.

#### 3.2.3 General self-efficacy scale (GSE)

To meet the needs of this study, the GSE was adapted to assess college students' SE in engaging in PE. The specific items and scoring details are presented in Table 3. Based on the original work of (Jerusalem and Schwarzer 2013), the scale has been extensively utilized and validated across various populations. The adapted scale in this study evaluates students' SE in PE across ten dimensions, such as persistence, resilience, and confidence in overcoming exercise-related challenges. It uses a 4-point rating system, with scores ranging from 1 (Not at all true) to 5 (Exactly true), where higher scores indicate greater SE. In this study, the Cronbach's alpha coefficient was 0.862.

**Table 3:** Self-efficacy scale.

**Serial number**	**Measurement items**
Item 1	I believe I can persist in achieving my exercise goals.
Item 2	Even when facing difficulties, I can find ways to continue my exercise routine.
Item 3	I am confident in my performance during physical exercise.
Item 4	I am convinced that I can handle unexpected challenges during physical exercise.
Item 5	I am confident that I can meet or exceed my expected goals in physical exercise.
Item 6	I believe my efforts will lead to progress in my physical exercise.
Item 7	I can remain calm during physical exercise, even under pressure.
Item 8	When faced with difficulties in physical exercise, I can usually come up with several solutions.
Item 9	I believe I have the ability to overcome obstacles in physical exercise.
Item 10	No matter what exercise tasks I encounter, I can handle them effectively.

To adapt the GSE scale for the context of physical exercise, we invited two psychology professors and two physical education professors to conduct a two-round content validity assessment of the scale. Their expertise ensured that the scale items were relevant and appropriate for measuring SE in the context of physical exercise. Based on this, we conducted a pilot study involving 90 college students, with 88 participants providing valid data. The pilot study aimed to test the comprehensibility of the scale items and to conduct preliminary reliability and validity analyses. The results showed that the scale met the required psychometric standards.

Subsequently, we proceeded with the formal survey. In the formal survey, we first conducted an EFA. The EFA results revealed a KMO value of 0.939, with one factor having an eigenvalue greater than 1, and the scree plot also indicated that a one-factor model was optimal. Building on this, we further conducted a CFA. The CFA results showed good model fit indices: χ^2^/*df* = 1.968, GFI = 0.995, AGFI = 0.992, TLI = 0.994, CFI = 0.995, and RMSEA = 0.019. These indices collectively demonstrated the scale's strong reliability and validity.

#### 3.2.4 Physical exercise

PE was assessed via a single question: “Over the past 7 days, how many days did you engage in at least 20 min of PE or activity that made you sweat or breathe heavily?” Participants could respond with a number ranging from 0 to 7 days. This method of assessment has been used in previous studies (Wang et al., 2025; Uddin et al., 2020; Roberts et al., 2024; Huang et al., 2025).

### 3.3 Statistical analysis

Prior to the primary analyses, to ensure the normality of the data, we excluded data points with absolute skewness greater than 1 and kurtosis greater than 3 by standard statistical practices. In conducting the data analysis, we employed SPSS 26.0. We began by computing descriptive statistics for the demographic variables of age and gender, as well as for the scale scores related to Grit, PGI, SE, and PE. Following this, we utilized Pearson correlations to explore the interrelationships among these variables. The normality of the scale score distributions was evaluated using skewness and kurtosis tests. Subsequently, we carried out mediation analysis with the SPSS PROCESS macro (Model 6) to delve into how PGI and SE mediate the relationship between Grit and PE (Hayes et al., 2017). For estimating indirect effects, we applied the bias-corrected Bootstrap method, utilizing 5000 bootstrap samples. A 95% confidence interval that excluded zero was considered indicative of a significant mediating effect (Cheung and Lau, 2008). Prior to conducting the mediation analysis, all predictor, mediator, and outcome variables were standardized (i.e., converted to Z-scores) to facilitate the interpretation of the path coefficients. In this analysis, gender and family residence were incorporated as covariates.

## 4 Results

### 4.1 Common method bias test

To assess the potential impact of standard method bias stemming from self-reported data, we employed Harman's single-factor test. The initial factor accounted for 19.74% of the variance, which is considerably lower than the 40% cutoff (Podsakoff et al., 2003). This suggests that common method bias does not pose a substantial concern in this study.

### 4.2 Descriptive statistics

Table 4 presents the fundamental descriptive statistics for Grit, PGI, SE, and PE. The mean scores were 3.376 ± 0.957 for Grit, 3.035 ± 0.681 for PGI, 2.718 ± 0.697 for SE, and 2.940 ± 1.903 for PE. To evaluate the normality of these variables' distributions, skewness and kurtosis tests were performed. With a sample size over 300, variables having skewness below three and kurtosis below eight are deemed approximately normally distributed (Kim, 2013). As indicated in Table 4, the skewness values fall between −0.228 and 0.378, and the kurtosis values are between −0.964 and −0.138, suggesting near-normal distributions. All variables showed significant correlations. Grit correlated positively with PGI (*r* = 0.410, *P* < 0.01), SE (*r* = 0.390, *P* < 0.01), and PE (*r* = 0.315, *P* < 0.01). PGI correlated positively with SE (*r* = 0.238, *P* < 0.01) and PE (*r* = 0.288, *P* < 0.01). SE also correlated positively with PE (*r* = 0.345, *P* < 0.01).

**Table 4:** Descriptive statistics and correlation matrix.

**Variables**	***M***	***SD***	***Skewness***	***Kurtosis***	**1**	**2**	**3**	**4**
1. Grit	3.376	0.957	−0.228	−0.964	1			
2. Personal growth initiative	3.035	0.681	−0.197	−0.138	0.410^***^	1		
3. Self-efficacy	2.718	0.697	−0.278	−0.895	0.390^***^	0.238^***^	1	
4. Physical exercise	2.940	1.903	0.378	−0.577	0.315^***^	0.288^***^	0.345^***^	1

^***^P < 0.001.

### 4.3 Chain mediation effect analysis

**Figure 2:**
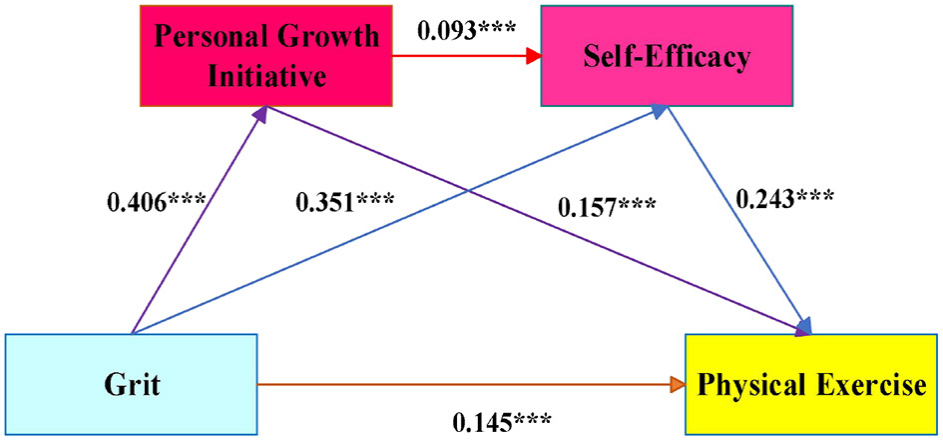
Structural equation model. *P < 0.05, **P < 0.01, ***P < 0.001.

After standardizing the four variables and controlling for gender and family residence, mediation analysis was conducted using Model 6, with the results shown in Table 5. Grit was found to be positively correlated with PGI (β = 0.406, *P* < 0.001), SE (β = 0.351, *P* < 0.001), and PE (β = 0.145, *P* < 0.001). PGI was positively correlated with SE (β = 0.093, *P* < 0.001) and PE (β = 0.157, *P* < 0.001). Moreover, SE was positively correlated with PE (β = 0.243, *P* < 0.001). The tested model diagram is illustrated in Figure 2.

**Table 5:** Regression analysis results.

**Dependent variable**	**Independent variable**	**β**	***SE***	***t***	***R^2^***	***F***
Personal growth initiative	Gender	−0.094	0.036	−2.615^**^	0.171	176.12^***^
	Family residence	0.074	0.037	2.030^*^		
	Grit	0.406	0.018	22.501^***^		
Self-efficacy	Gender	−0.034	0.036	−0.936	0.160	121.324^***^
	Family residence	0.005	0.037	0.135		
	Grit	0.351	0.020	17.621^***^		
	Personal growth initiative	0.093	0.020	4.660^***^		
Physical exercise	Gender	−0.315	0.035	−8.915^***^	0.210	135.433^***^
	Family residence	0.134	0.036	3.760^***^		
	Grit	0.145	0.020	7.069^***^		
	Personal growth initiative	0.157	0.019	8.069^***^		
	Self-efficacy	0.243	0.019	12.675^***^		

^*^P < 0.05, ^**^P < 0.01, ^***^P < 0.001.

The study utilized Hayes' bias-corrected non-parametric percentile Bootstrap method, employing 5000 bootstrap samples to determine the 95% confidence intervals. Mediating effects were deemed statistically significant if their 95% confidence intervals excluded zero (Hayes et al., 2017). As shown in Table 6, significant mediating effects were identified for PGI (95% CI: [0.047, 0.081], effect size: 0.064), SE (95% CI: [0.070, 0.102], effect size: 0.085), and the chain mediation of PGI and SE (95% CI: [0.005, 0.014], effect size: 0.009). These results confirm that all three pathways significantly mediate the relationship between Grit and college students' PE, thereby validating the study's hypotheses. It is important to note that this method does not report fit indices such as RMSEA (Preacher and Hayes, 2008).

**Table 6:** Mediation effects and proportions.

**Path**	**Effect**	**Boot SE**	**Boot LLCI**	**Boot ULCI**	**Effect proportion**
Total effect	0.303	0.018	0.266	0.339	100%
Direct effect	0.145	0.020	0.104	0.185	48%
Total indirect effect	0.158	0.011	0.138	0.180	52%
Ind1	0.064	0.009	0.047	0.081	21%
Ind2	0.085	0.008	0.070	0.102	28%
Ind3	0.009	0.002	0.005	0.014	3%

This table outlines the direct and indirect impacts of Grit, PGI, and SE on college students' PE. The Boot SE, Boot LLCI, and Boot ULCI indicate the standard error and the 95% confidence interval limits, respectively, calculated via the bias-corrected percentile bootstrap approach. Ind1: Grit → PGI → PE; Ind2: Grit → SE → PE; Ind3: Grit → PGI → SE → PE.

## 5 Discussion

The current investigation has identified a significant positive correlation between college students' Grit and their PE. PGI and SE not only function as independent mediators but also collectively form a chain mediation effect. These findings not only offer a fresh perspective on how psychological traits in college students influence healthy behaviors but also provide a scientific basis for colleges to design interventions targeting mental health and PE. Theoretically, this model advances our understanding of the psychological mechanisms linking dispositional traits to health behaviors. Practically, it provides a valuable blueprint for designing effective health promotion interventions in university settings, shifting the focus from simply encouraging activity to cultivating the underlying psychological attributes that sustain it.

### 5.1 The impact of grit on physical exercise among college students

The results of this study indicate a significant positive correlation between Grit and PE among college students, a finding that aligns with prior research and confirms hypothesis 1. This conclusion is similar to previous research findings (Kim, 2020; Messina et al., 2020; Guelmami et al., 2022; Liu et al., 2025).

Individuals with high levels of Grit likely possess the resilience to overcome common barriers to exercise, such as time constraints or fatigue. Additionally, when students set exercise goals that match their skill levels, they experience a sense of accomplishment, which ignites interest, reduces the fear of failure, and strengthens their motivation to continue participating in physical activities (Jacobs et al., 1984; Sidenko, 2025). At the same time, we must consider alternative explanations for this correlation. It is possible that confounding factors, such as pre-existing levels of social support, access to university sports facilities, or even underlying genetic predispositions for activity, could influence both an individual's Grit and their exercise habits. Furthermore, the relationship could be reciprocal, with regular exercise itself helping to build discipline and Grit over time.

Conversely, students lacking Grit often struggle to maintain a positive mindset when confronted with challenges in PE and are more likely to give up when encountering difficulties (Gorin et al., 2024). This behavior not only undermines their self-confidence but also decreases their likelihood of continuing to engage in PE. Therefore, cultivating Grit in college students is crucial for increasing their participation and persistence in PE. By enhancing students' Grit, we can help them maintain a positive attitude when facing difficulties in PE, thereby improving their participation and exercise outcomes. However, it is crucial to acknowledge that our cross-sectional design only establishes an association; it does not permit causal claims. Future longitudinal research is needed to confirm whether cultivating Grit indeed leads to an increase in physical exercise.

### 5.2 The independent mediating role of personal growth initiative

This study confirms that college students' Grit not only directly promotes PE but also indirectly influences this behavior through PGI, thereby supporting hypothesis 2. This finding is consistent with the Organic Assessment Process Theory (Maurer and Daukantaite, 2020; Li et al., 2023) and Goal-Setting Theory (Locke and Latham, 2006), offering a new perspective for understanding the PE of college students. The Organic Assessment Process Theory posits that an individual's clear understanding of their own abilities helps them assess the potential value of new stimuli, thereby facilitating self-actualization (Tao et al., 2024a). In the context of PE among college students, this theory underscores the pivotal role of self-awareness in fostering PE. College students with higher levels of Grit typically possess a clearer understanding of their abilities, enabling them to better evaluate the value of PE and regard it as a vital pathway for self-improvement (Liu M. et al., 2024; Longakit et al., 2025). Goal-setting theory further elucidates this process (Locke and Latham, 2019). This theory posits that clear and challenging goals can significantly enhance an individual's motivation and behavioral performance. College students with high Grit are more inclined to set challenging PE goals and translate these goals into concrete actions through PGI (Cai and Lian, 2022; Yue et al., 2025; Weigold et al., 2024). This goal-setting process not only bolsters their intrinsic motivation but also heightens their persistence in the face of difficulties, thereby sustaining their PE. Empirical studies have also confirmed that PGI, as a core driving force for self-improvement and goal achievement, can effectively transform Grit into concrete actions. College students with high PGI are more likely to proactively engage in behaviors that benefit themselves (Lin et al., 2024; Weigold et al., 2024; Çankaya et al., 2017).

Therefore, universities should encourage students' PGI. This can be achieved through concrete applications. For instance, universities could offer workshops focused on developing self-awareness, personal goal-setting, and reflective practices. Another promising approach is curriculum integration, where personal development modules could be embedded within first-year orientation programs or general education courses, guiding students to actively plan their academic, personal, and health-related growth. However, implementing such interventions is not without challenges. Obstacles may include low student engagement due to academic pressures, a lack of trained facilitators, and limited institutional resources. To overcome these hurdles, universities could offer incentives such as course credits or micro-credentials for participation. Leveraging peer mentors trained in PGI principles could offer a scalable and relatable solution, while partnerships with university counseling centers can provide the necessary professional expertise.

### 5.3 The independent mediating role of self-efficacy

The study results confirmed hypothesis 3, that SE acts as an independent mediator between Grit and college students' PE. This aligns with Bandura's Self-Efficacy theory (Bandura, 2001), which posits that individuals' beliefs and cognitive mechanisms significantly influence their behavior and psychological states (Bandura, 1982). As a significant psychological trait, Grit can notably affect college students' SE, which in turn impacts their PE (DuCharme and Brawley, 1995; Zhou and Huo, 2022). This finding is consistent with numerous prior studies, regardless of whether qualitative or quantitative methods were used and whether the samples were from Western or Eastern contexts (Jacobs et al., 1984; Bill et al., 2023; Liu L. et al., 2024; Judge and Bono, 2001; Mesurado et al., 2018). It is important, however, to consider the nuances of measuring self-efficacy. Contextual factors can influence self-reported efficacy.

Prior research has indicated that individuals with high SE tend to employ more positive and effective strategies when confronting challenges (Bill et al., 2023; Yu et al., 2025). As a result, they generally have better experiences within the realm of PE and health. Given the mutually reinforcing relationship between PE and health status, one might wonder whether a similar mechanism and pattern of action exists between college students' SE and health status. To explore this dynamic interplay, future research should move beyond cross-sectional designs. A longitudinal approach, such as a cross-lagged panel model, would be ideal for disentangling the temporal precedence and estimating the strength of the reciprocal effects between SE and health status over an academic year.

Overall, SE plays a crucial role in college students' PE. It is not only a vital mediating mechanism through which Grit influences PE but also a key psychological factor that enables college students to maintain a positive attitude and continue participating in PE. This finding underscores the importance of cultivating SE among college students. In future educational practices, colleges and relevant educational institutions should place great emphasis on fostering college students' SE. By designing and implementing targeted educational interventions, such as offering psychological counseling courses related to PE, organizing a variety of sports competitions, and providing personalized PE guidance, these institutions can help college students develop a proper concept of PE, enhance their SE, and thus promote their active participation in PE. This will improve their physical and mental health and lay a solid foundation for their comprehensive development.

### 5.4 The chain mediating role of personal growth initiative and self-efficacy

The study results confirmed hypothesis 4, that PGI and SE act as a chain mediator between Grit and PE among college students. This complete pathway, as visualized in our proposed model (see Figure 1). On the one hand, Grit is an important psychological quality in the personal development and growth of college students (Hou et al., 2022). The proactive PGI cultivated and shaped by Grit is crucial for the development of PE in college students. College students with Grit exhibit stronger PGI; they are more willing to actively explore new ways and opportunities for PE and to seek out exercise plans and goals that suit them (Yu et al., 2025; Zhao and Yin, 2024). This proactive attitude enables them to continually try and challenge themselves during PE, thereby gaining more exercise experience and successful experiences (Caspersen et al., 1985). These successful experiences and positive feedback are important sources of SE. Therefore, college students with higher levels of PGI often have higher levels of SE, and vice versa (Weigold et al., 2024).

On the other hand, college students with high PGI receive more attention and recognition during PE. In the campus environment, where sports resources are relatively limited, those with high PGI participate more actively in sports activities, demonstrating their athletic abilities and enthusiasm for PE (Yukhymenko-Lescroart, 2021; Almagro et al., 2020). This active participation and demonstration attract more attention and affirmation, including from peers, coaches, and sports clubs. This attention and affirmation create a psychological advantage that enhances their SE. In summary, Grit, PGI, and SE are key factors influencing the PE of college students.

Although all mediation paths in our study are statistically significant, the chain mediating effect (PGI → SE) accounts for only 3% of the total effect. This small effect size may reflect the complex psychological mechanisms between PGI and SE, which various factors could moderate. Nevertheless, the identification of this chain mediating path holds significant theoretical importance. It reveals the underlying mechanism through which Grit influences physical exercise behavior via PGI and SE, offering a new perspective on how psychological processes mediate the impact of Grit on healthy behaviors. From a practical standpoint, this path underscores the importance of fostering personal growth, initiative, and self-efficacy. By enhancing students' PGI, we can indirectly boost their self-efficacy, thereby promoting physical exercise behavior. This intervention strategy may be particularly effective for students who lack intrinsic motivation. Future research should further explore the relationship between PGI and SE, as well as their roles in different cultural and educational contexts, to gain a more comprehensive understanding of how Grit affects physical exercise behavior.

### 5.5 Limitations

This study has several limitations that should be acknowledged. First, the assessment of PE was primarily conducted through a single-item self-report measure, without the use of objective assessment tools such as accelerometers or fitness trackers. Although self-reporting is commonly used in research due to its feasibility and cost-effectiveness, it is subject to recall bias and social desirability bias, which may affect the accuracy of participants' reports regarding their PE levels, potentially influencing the study's findings. Second, the relationship between Grit, PGI, SE, and PE is complex and likely influenced by multiple factors, including individual differences, environmental factors, and cultural contexts. However, the cross-sectional design of this study limits the ability to infer causal relationships among these variables. Future studies using longitudinal designs are needed to better elucidate the temporal and causal relationships among Grit, PGI, SE, and PE. Third, the study sample consisted exclusively of college students from central China, which may limit the generalizability of the findings to other populations. Future research should include more diverse cohorts, such as students from different regions, different educational levels, and different cultural backgrounds, to determine whether the observed associations among Grit, PGI, SE, and PE hold across different demographic and cultural contexts. Fourth, this study used a convenience sampling method from six universities in central China, which may cause insufficient representation of students from other regions or types of educational institutions. Future studies are suggested to expand the sampling range to include a broader and more diverse sample of college students, in order to enhance the external validity of the findings.

## 6 Conclusion

This study thoroughly explored the correlation between Grit and PE among college students, with a particular focus on the chain mediating effect of PGI and SE. The results showed a significant positive correlation between Grit and PE. PGI and SE were identified as key mediators in this relationship, with PGI fostering the development of SE, which subsequently promotes PE. These findings underscore the importance of prioritizing the assessment and management of Grit, PGI, and SE among college students.To promote positive experiences among college students, educational practitioners and relevant professionals are encouraged to implement targeted strategies for early identification, prevention, and intervention. For instance, universities might organize specialized mental health education activities, including workshops, lectures, and counseling sessions, aimed at helping students enhance their Grit, PGI, and SE. Moreover, there is a pressing need for qualitative and longitudinal research, as well as surveys targeting college students, to evaluate their awareness of these psychological qualities. Such efforts will contribute to a deeper understanding of the complex interrelationships involved and the generation of robust, systematic evidence.

## Data Availability

The original contributions presented in the study are included in the article/Supplementary material, further inquiries can be directed to the corresponding author.
