# Supplementary material for: How grit enhances physical exercise in college students: mediating roles of personal growth initiative and self-efficacy
Source: Front Psychol. 2025 Sep 9;16:1652984. doi: 10.3389/fpsyg.2025.1652984 (PMC12455857; doi:10.3389/fpsyg.2025.1652984)
Supplement: Supplementary file 1 [file Supplementary_file_1.docx]

# Supplementary Material

1、 Reliability analysis

The questionnaires used in this study have been thoroughly validated for reliability and validity. However, the study still conducted an analysis. The results are as shown in Table 1. The reliability of the variables in this study ranges from 0.753 to 0.862. Specifically, the reliability of Grit is 0.849 with 8 items, the reliability of Personal growth initiative is 0.753 with 16 items, and the reliability of Self-efficacy is 0.862 with 10 items. These results indicate that the research scales and variables have a relatively high degree of reliability, demonstrating good stability and consistency.

S-Table 1 Reliability analysis results

|  | Alpha | Item number |
| --- | --- | --- |
| Grit | 0.849 | 8 |
| Personal growth initiative | 0.753 | 16 |
| Self-efficacy | 0.862 | 10 |

1. **EFA**
2. **Grit**

S-Table 2 KMO and Bartlett's Test

| Kaiser-Meyer-Olkin Measure of Sampling Adequacy. |  | 0.889 |
| --- | --- | --- |
| Bartlett's Test of Sphericity | Approx. Chi-Square | 6975.772 |
|  | df | 28 |
|  | Sig. | 0 |

S-Table 4 Communalities

|  | Initial | Extraction |
| --- | --- | --- |
| X1 | 1 | 0.644 |
| X2 | 1 | 0.621 |
| X3 | 1 | 0.617 |
| X4 | 1 | 0.632 |
| X5 | 1 | 0.65 |
| X6 | 1 | 0.604 |
| X7 | 1 | 0.637 |
| X8 | 1 | 0.619 |

S-Table 5 Total Variance Explained

| Component | Initial Eigenvalues |  |  | Extraction Sums of Squared Loadings |  |  | Rotation Sums of Squared Loadings |  |  |
| --- | --- | --- | --- | --- | --- | --- | --- | --- | --- |
|  | Total | % of Variance | Cumulative % | Total | % of Variance | Cumulative % | Total | % of Variance | Cumulative % |
| 1 | 3.89 | 48.619 | 48.619 | 3.89 | 48.619 | 48.619 | 2.514 | 31.428 | 31.428 |
| 2 | 1.135 | 14.184 | 62.803 | 1.135 | 14.184 | 62.803 | 2.51 | 31.375 | 62.803 |
| 3 | 0.541 | 6.757 | 69.559 |  |  |  |  |  |  |
| 4 | 0.511 | 6.382 | 75.941 |  |  |  |  |  |  |
| 5 | 0.498 | 6.223 | 82.164 |  |  |  |  |  |  |
| 6 | 0.491 | 6.142 | 88.306 |  |  |  |  |  |  |
| 7 | 0.475 | 5.94 | 94.246 |  |  |  |  |  |  |
| 8 | 0.46 | 5.754 | 100 |  |  |  |  |  |  |

S-Table 6 Rotated Component Matrixa

|  | Component |  |
| --- | --- | --- |
|  | 1 | 2 |
| X1 | 0.771 |  |
| X2 | 0.756 |  |
| X3 | 0.747 |  |
| X4 | 0.764 |  |
| X5 |  | 0.776 |
| X6 |  | 0.736 |
| X7 |  | 0.770 |
| X8 |  | 0.753 |

1. **personal growth initiative**

S-Table 7 KMO and Bartlett's Test

| Kaiser-Meyer-Olkin Measure of Sampling Adequacy. |  | 0.807 |
| --- | --- | --- |
| Bartlett's Test of Sphericity | Approx. Chi-Square | 7690.913 |
|  | df | 120 |
|  | Sig. | 0 |

S-Table 8 Communalities

|  | Initial | Extraction |
| --- | --- | --- |
| m1 | 1 | 0.483 |
| m2 | 1 | 0.476 |
| m3 | 1 | 0.51 |
| m4 | 1 | 0.518 |
| m5 | 1 | 0.518 |
| m6 | 1 | 0.511 |
| m7 | 1 | 0.484 |
| m8 | 1 | 0.518 |
| m9 | 1 | 0.507 |
| m10 | 1 | 0.601 |
| m11 | 1 | 0.612 |
| m12 | 1 | 0.579 |
| m13 | 1 | 0.597 |
| m14 | 1 | 0.545 |
| m15 | 1 | 0.551 |
| m16 | 1 | 0.58 |

S-Table 9 Total Variance Explained

| Component | Initial Eigenvalues |  |  | Extraction Sums of Squared Loadings |  |  | Rotation Sums of Squared Loadings |  |  |
| --- | --- | --- | --- | --- | --- | --- | --- | --- | --- |
|  | Total | % of Variance | Cumulative % | Total | % of Variance | Cumulative % | Total | % of Variance | Cumulative % |
| 1 | 3.438 | 21.489 | 21.489 | 3.438 | 21.489 | 21.489 | 2.539 | 15.87 | 15.87 |
| 2 | 1.906 | 11.91 | 33.398 | 1.906 | 11.91 | 33.398 | 2.273 | 14.209 | 30.079 |
| 3 | 1.746 | 10.911 | 44.31 | 1.746 | 10.911 | 44.31 | 1.99 | 12.438 | 42.518 |
| 4 | 1.5 | 9.377 | 53.687 | 1.5 | 9.377 | 53.687 | 1.787 | 11.169 | 53.687 |
| 5 | 0.749 | 4.678 | 58.365 |  |  |  |  |  |  |
| 6 | 0.706 | 4.413 | 62.778 |  |  |  |  |  |  |
| 7 | 0.676 | 4.226 | 67.004 |  |  |  |  |  |  |
| 8 | 0.652 | 4.072 | 71.076 |  |  |  |  |  |  |
| 9 | 0.637 | 3.981 | 75.057 |  |  |  |  |  |  |
| 10 | 0.617 | 3.855 | 78.911 |  |  |  |  |  |  |
| 11 | 0.611 | 3.818 | 82.729 |  |  |  |  |  |  |
| 12 | 0.591 | 3.695 | 86.424 |  |  |  |  |  |  |
| 13 | 0.581 | 3.63 | 90.054 |  |  |  |  |  |  |
| 14 | 0.545 | 3.403 | 93.457 |  |  |  |  |  |  |
| 15 | 0.53 | 3.312 | 96.769 |  |  |  |  |  |  |
| 16 | 0.517 | 3.231 | 100 |  |  |  |  |  |  |

S-Table 10 Total Variance Explained

|  | Component |  |  |  |
| --- | --- | --- | --- | --- |
|  | 1 | 2 | 3 | 4 |
| m1 |  |  | 0.688 |  |
| m2 |  |  | 0.685 |  |
| m3 |  |  | 0.703 |  |
| m4 |  |  | 0.706 |  |
| m5 | 0.708 |  |  |  |
| m6 | 0.704 |  |  |  |
| m7 | 0.688 |  |  |  |
| m8 | 0.713 |  |  |  |
| m9 | 0.706 |  |  |  |
| m10 |  |  |  | 0.764 |
| m11 |  |  |  | 0.773 |
| m12 |  |  |  | 0.748 |
| m13 |  | 0.768 |  |  |
| m14 |  | 0.723 |  |  |
| m15 |  | 0.732 |  |  |
| m16 |  | 0.754 |  |  |

1. **self-efficacy**

S-Table 11 KMO and Bartlett's Test

| Kaiser-Meyer-Olkin Measure of Sampling Adequacy. |  | 0.939 |
| --- | --- | --- |
| Bartlett's Test of Sphericity | Approx. Chi-Square | 7420.744 |
|  | df | 45 |
|  | Sig. | 0 |

S-Table 12 Communalities

|  | Initial | Extraction |
| --- | --- | --- |
| z1 | 1 | 0.432 |
| z2 | 1 | 0.453 |
| z3 | 1 | 0.465 |
| z4 | 1 | 0.429 |
| z5 | 1 | 0.453 |
| z6 | 1 | 0.474 |
| z7 | 1 | 0.471 |
| z8 | 1 | 0.425 |
| z9 | 1 | 0.44 |
| z10 | 1 | 0.42 |

S-Table 13Total Variance Explained

| Component | Initial Eigenvalues |  |  | Extraction Sums of Squared Loadings |  |  |
| --- | --- | --- | --- | --- | --- | --- |
|  | Total | % of Variance | Cumulative % | Total | % of Variance | Cumulative % |
| 1 | 4.462 | 44.619 | 44.619 | 4.462 | 44.619 | 44.619 |
| 2 | 0.705 | 7.047 | 51.666 |  |  |  |
| 3 | 0.673 | 6.728 | 58.393 |  |  |  |
| 4 | 0.648 | 6.481 | 64.875 |  |  |  |
| 5 | 0.624 | 6.242 | 71.117 |  |  |  |
| 6 | 0.612 | 6.118 | 77.235 |  |  |  |
| 7 | 0.601 | 6.007 | 83.242 |  |  |  |
| 8 | 0.583 | 5.831 | 89.073 |  |  |  |
| 9 | 0.561 | 5.606 | 94.679 |  |  |  |
| 10 | 0.532 | 5.321 | 100 |  |  |  |

S-Table 14 Component Matrixa

|  | Component |
| --- | --- |
|  | 1 |
| z1 | 0.657 |
| z2 | 0.673 |
| z3 | 0.682 |
| z4 | 0.655 |
| z5 | 0.673 |
| z6 | 0.688 |
| z7 | 0.686 |
| z8 | 0.652 |
| z9 | 0.663 |
| z10 | 0.648 |

**2、CFA**

**1） Grit**


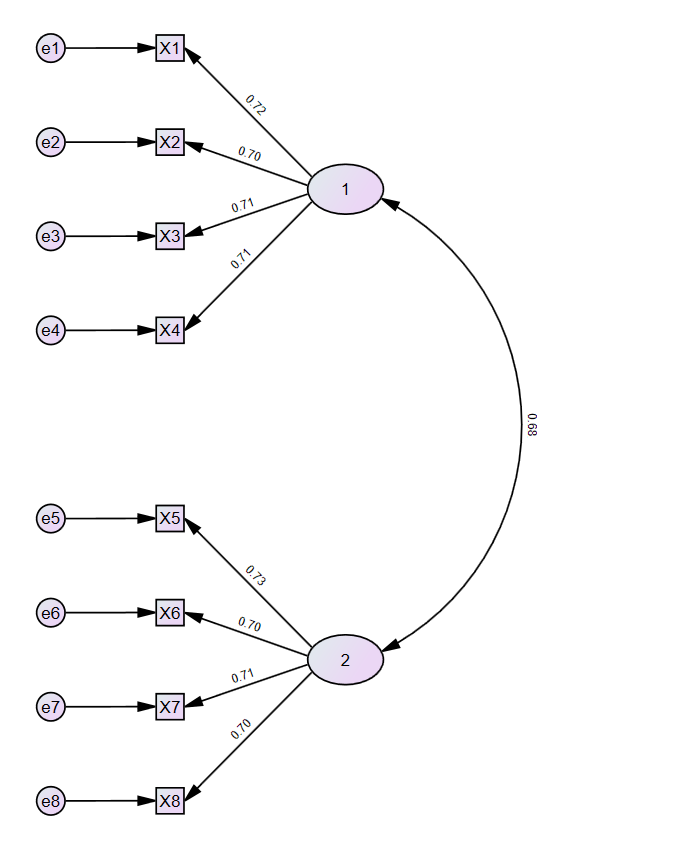


S-Table 15 Model Fit Results

|  | Reasonable Standard | Excellent Standard | Model Valu | Judgment | result |
| --- | --- | --- | --- | --- | --- |
| CMIN | 19.051 | | | | |
| CMIN/DF | <5 | <3 | 1.003 | Excellent | accept |
| GFI | >0.8 | >0.9 | 0.998 | Excellent | accept |
| AGFI | >0.8 | >0.9 | 0.996 | Excellent | accept |
| NFI | >0.8 | >0.9 | 0.997 | Excellent | accept |
| IFI | >0.8 | >0.9 | 1 | Excellent | accept |
| TLI | >0.8 | >0.9 | 1 | Excellent | accept |
| CFI | >0.8 | >0.9 | 1 | Excellent | accept |
| RMSEA | <0.08 | <0.05 | 0.001 | Excellent | accept |

It can be seen from the s-table 15: the model parameters all meet the reasonable standards, indicating that the model fit is up to the mark, and the model is acceptable.

**2）personal growth initiative**


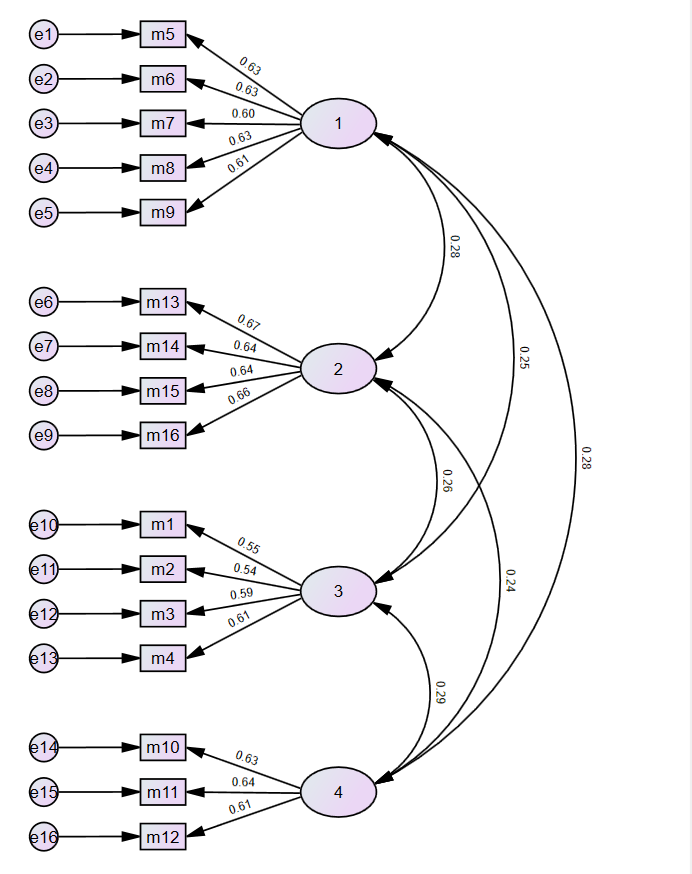


S-Table 16Model Fit Results

|  | Reasonable Standard | Excellent Standard | Model Valu | Judgment | result |
| --- | --- | --- | --- | --- | --- |
| CMIN | 159.490 | | | | |
| CMIN/DF | <5 | <3 | 1.627 | Reasonable | accept |
| GFI | >0.8 | >0.9 | 0.992 | Excellent | accept |
| AGFI | >0.8 | >0.9 | 0.989 | Excellent | accept |
| NFI | >0.8 | >0.9 | 0.979 | Excellent | accept |
| IFI | >0.8 | >0.9 | 0.992 | Excellent | accept |
| TLI | >0.8 | >0.9 | 0.990 | Excellent | accept |
| CFI | >0.8 | >0.9 | 0.992 | Excellent | accept |
| RMSEA | <0.08 | <0.05 | 0.016 | Reasonable | accept |

It can be seen from the s-table 16: the model parameters all meet the reasonable standards, indicating that the model fit is up to the mark, and the model is acceptable.

**3）self-efficacy**


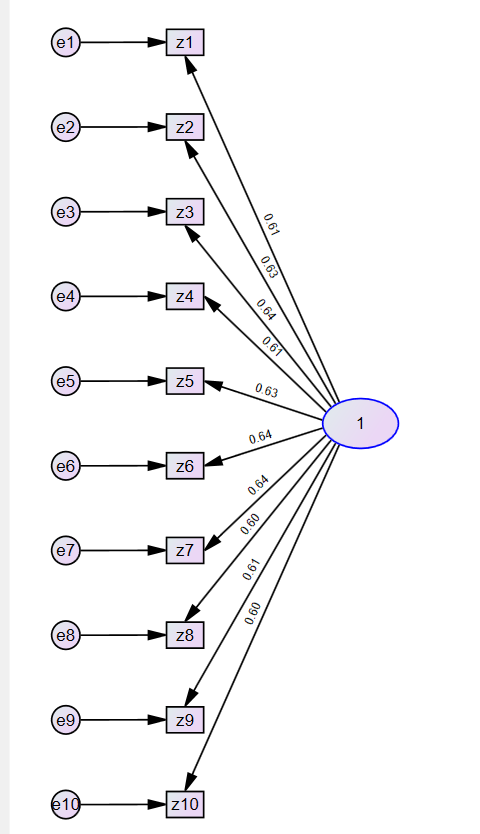


S-Table 17 Model Fit Results

|  | Reasonable Standard | Excellent Standard | Model Valu | Judgment | result |
| --- | --- | --- | --- | --- | --- |
| CMIN | 68.890 | | | | |
| CMIN/DF | <5 | <3 | 1.968 | Excellent | accept |
| GFI | >0.8 | >0.9 | 0.995 | Excellent | accept |
| AGFI | >0.8 | >0.9 | 0.992 | Excellent | accept |
| NFI | >0.8 | >0.9 | 0.991 | Excellent | accept |
| IFI | >0.8 | >0.9 | 0.995 | Excellent | accept |
| TLI | >0.8 | >0.9 | 0.994 | Excellent | accept |
| CFI | >0.8 | >0.9 | 0.995 | Excellent | accept |
| RMSEA | <0.08 | <0.05 | 0.019 | Excellent | accept |

It can be seen from the s-table 17: the model parameters all meet the reasonable standards, indicating that the model fit is up to the mark, and the model is acceptable.

| S-Table 8 Grade | | | | | |
| --- | --- | --- | --- | --- | --- |
|  | | Frequence | % | Available% | Cumulative% |
| Valid | 7th Grade | 216 | 21.4 | 21.4 | 21.4 |
|  | 8th Grade | 202 | 20.0 | 20.0 | 41.4 |
|  | 9th Grade | 195 | 19.3 | 19.3 | 60.7 |
|  | 10th Grade | 209 | 20.7 | 20.7 | 81.4 |
|  | 11th Grade | 188 | 18.6 | 18.6 | 100.0 |
|  | Total | 1010 | 100.0 | 100.0 |  |
